# Applied artificial intelligence in dentistry: emerging data modalities and modeling approaches

**DOI:** 10.3389/frai.2024.1427517

**Published:** 2024-07-23

**Authors:** Balazs Feher, Camila Tussie, William V. Giannobile

**Affiliations:** ^1^Department of Oral Medicine, Infection, and Immunity, Harvard School of Dental Medicine, Boston, MA, United States; ^2^ITU/WHO/WIPO Global Initiative on Artificial Intelligence for Health, Geneva, Switzerland; ^3^Department of Oral Surgery, University Clinic of Dentistry, Medical University of Vienna, Vienna, Austria; ^4^Department of Oral Biology, University Clinic of Dentistry, Medical University of Vienna, Vienna, Austria

**Keywords:** artificial intelligence, machine learning, diagnostic modeling, prognostic modeling, generative modeling, dental medicine, dental radiography

## Abstract

Artificial intelligence (AI) is increasingly applied across all disciplines of medicine, including dentistry. Oral health research is experiencing a rapidly increasing use of machine learning (ML), the branch of AI that identifies inherent patterns in data similarly to how humans learn. In contemporary clinical dentistry, ML supports computer-aided diagnostics, risk stratification, individual risk prediction, and decision support to ultimately improve clinical oral health care efficiency, outcomes, and reduce disparities. Further, ML is progressively used in dental and oral health research, from basic and translational science to clinical investigations. With an ML perspective, this review provides a comprehensive overview of how dental medicine leverages AI for diagnostic, prognostic, and generative tasks. The spectrum of available data modalities in dentistry and their compatibility with various methods of applied AI are presented. Finally, current challenges and limitations as well as future possibilities and considerations for AI application in dental medicine are summarized.

## 1 Artificial intelligence in the medical context

**Figure 1:**
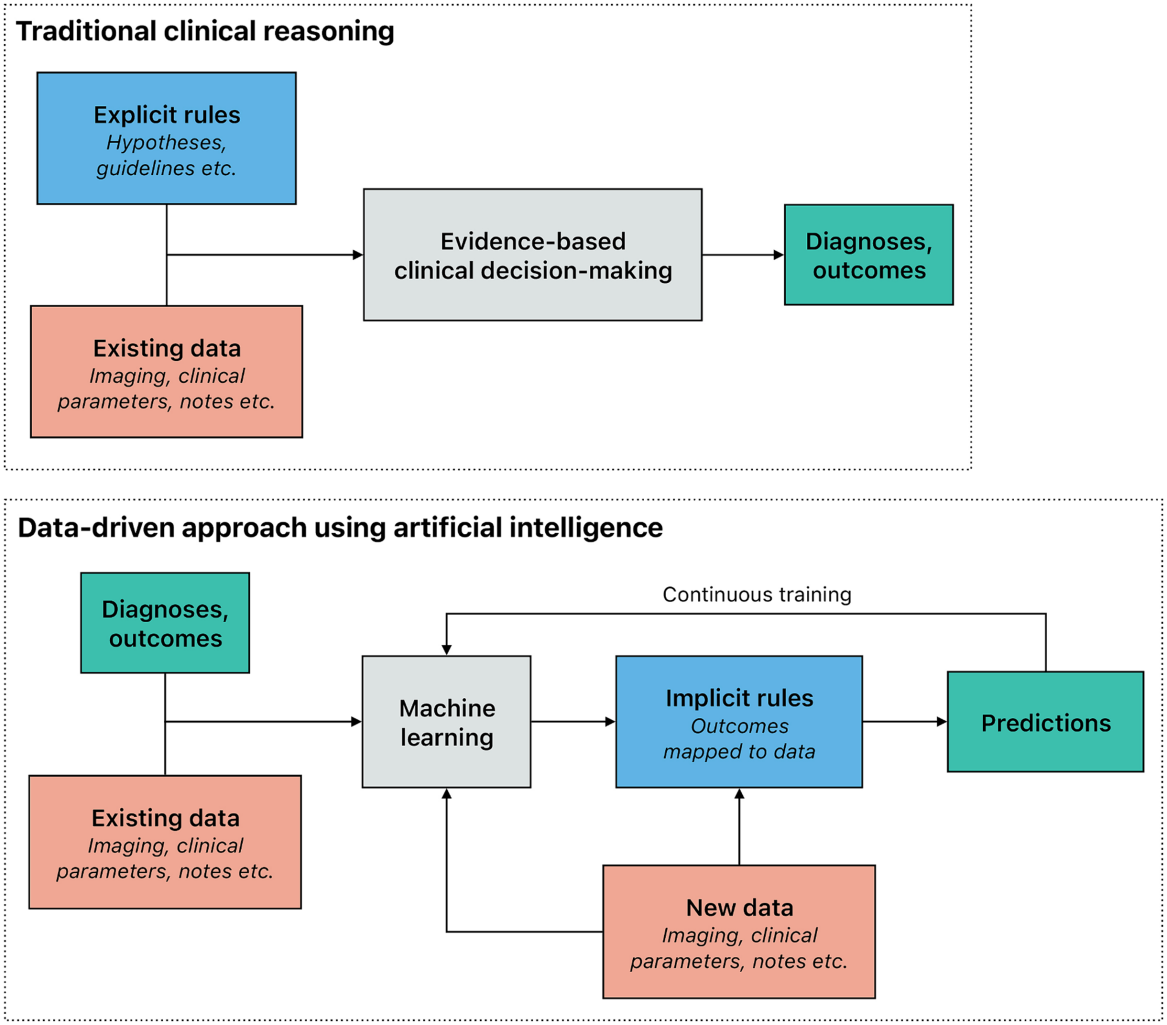
Traditional reasoning process compared to a data-driven approach using artificial intelligence in a clinical setting. In the traditional reasoning process within a clinical setting, explicit rules (e.g., those derived from pathophysiology) are applied to the observed data (e.g., a patient’s symptoms), to make a diagnosis or predict an outcome. Explicit rules should be based on scientific evidence. The traditional reasoning process is effective in situations where the underlying rules are straightforward and clear. However, it tends to fall short in more complex scenarios. In contrast, machine learning models, which are trained using numerous examples, infer implicit rules by correlating outcomes with the data. This data-driven approach not only adapts better to the presence of multicollinearity but also provides outcomes that are tailored to individual patients, making it particularly valuable for predictive modeling and personalized medicine. Machine learning models can be continuously improved as new data are acquired.

While the concept of artificial intelligence (AI), including its potential use in medicine, is several decades old (Turing, 1950), computing power remained a prohibitive bottleneck throughout most of the last century (Schwartz et al., 1987). Indeed, machine learning (ML), often considered the main branch of AI that identifies inherent patterns and connections in the input data similarly to how humans learn, is much more resource-intensive than most methods of classical statistics. Although classical statistics and ML are related fields, there is a key difference: ML models infer rules based on examples instead of being given explicit — if tentative — rules. Translated into clinical medicine, explicit rules describing straightforward causal relationships can be used for evidence-based decision making; nonetheless, complex patterns and interactions among numerous variables can be difficult to capture in this manner. In contrast, ML maps prior outcomes to the existing data to infer implicit rules (Figure 1). While this approach also requires more data than human learning and more processing power than classical statistics, ML models in turn can process massive datasets (Halevy et al., 2009), make predictions on new data, and use feedback for continuous training. The sustained, dramatic increase in semiconductor density — colloquially referred to as Moore’s law — and the availability of considerably larger datasets (i.e., big data) are two key factors that enabled the progressive development of ML. Big data and enhanced computing capabilities enable deep learning that leverages complex algorithms to model and understand intricate patterns in large volumes of data (Esteva et al., 2019; Topol, 2019). In today’s medicine, both deep learning and other forms of ML are increasingly used to augment the work of both clinicians and researchers (He et al., 2019).

Algorithms of applied ML in medicine can be categorized into three broad paradigms based on their learning style and by the extent to which input data are labeled: supervised, unsupervised, and semi-supervised learning. Supervised learning requires input data to be labeled as the algorithms learn from datasets that include both input data and corresponding correct outputs (e.g., radiographs used for training are first diagnosed by a human observer). In many cases, obtaining a strong supervisory signal (i.e., labeling of most or all of the dataset) is challenging. A combination of labeled and unlabeled input data enables weak supervision (Zhou, 2017). The promised advantage of weak supervision is the ability to process larger datasets while still not completely foregoing expert labeling. Unsupervised learning only uses unlabeled data as the algorithms try to find underlying patterns of structures using methods including clustering (i.e., grouping of similar instances) or dimensionality reduction (i.e., data simplification while retaining structure). Biomedical research uses unsupervised learning to identify previously unknown connections in an agnostic manner (e.g., protein structure prediction). Reinforcement learning uses unlabeled data to optimize a sequence of decisions in order to maximize a pre-set outcome (Sutton and Barto, 2018). Based on their use cases in medicine, most ML models can also broadly be categorized based on their task, variables, and output into regression and classification models. Regression models can be used to predict continuous outcomes. In medicine, this often includes quantifying the risk of an event or predicting the outcome of an intervention. Classification models can be used to categorize data into predefined classes. In the medical context, this includes establishing preliminary diagnoses or stratifying patients into risk categories. Computer vision, arguably one of the most widespread methods of ML in medicine, uses a combination of both classification and regression models to locate and interpret structures in image data.

Dental medicine has seen a swift increase in AI application comparable to other medical disciplines (Schwendicke et al., 2021c). Research output, clinical application, as well as public interest are increasing considerably. Notably, use and reporting of AI methods in dental medicine, including quantitative performance metrics, are characterized by a substantial heterogeneity. In the succeeding sections, a comprehensive overview of how various AI methods, including diagnostic, prognostic, and generative modeling used in dental medicine, is presented. Notably, several recent reviews have discussed the application of AI-enabled tools depending on different dental disciplines (e.g., periodontology, operative dentistry, oral radiology, etc.) (Nguyen et al., 2021a; Ding et al., 2023). In contrast to previous work, this article adopts an ML perspective, providing a cross-section of the spectrum of available data modalities, and exploring their compatibility with various AI methodologies.

### 1.1 Data modalities in dental medicine

**Figure 2:**
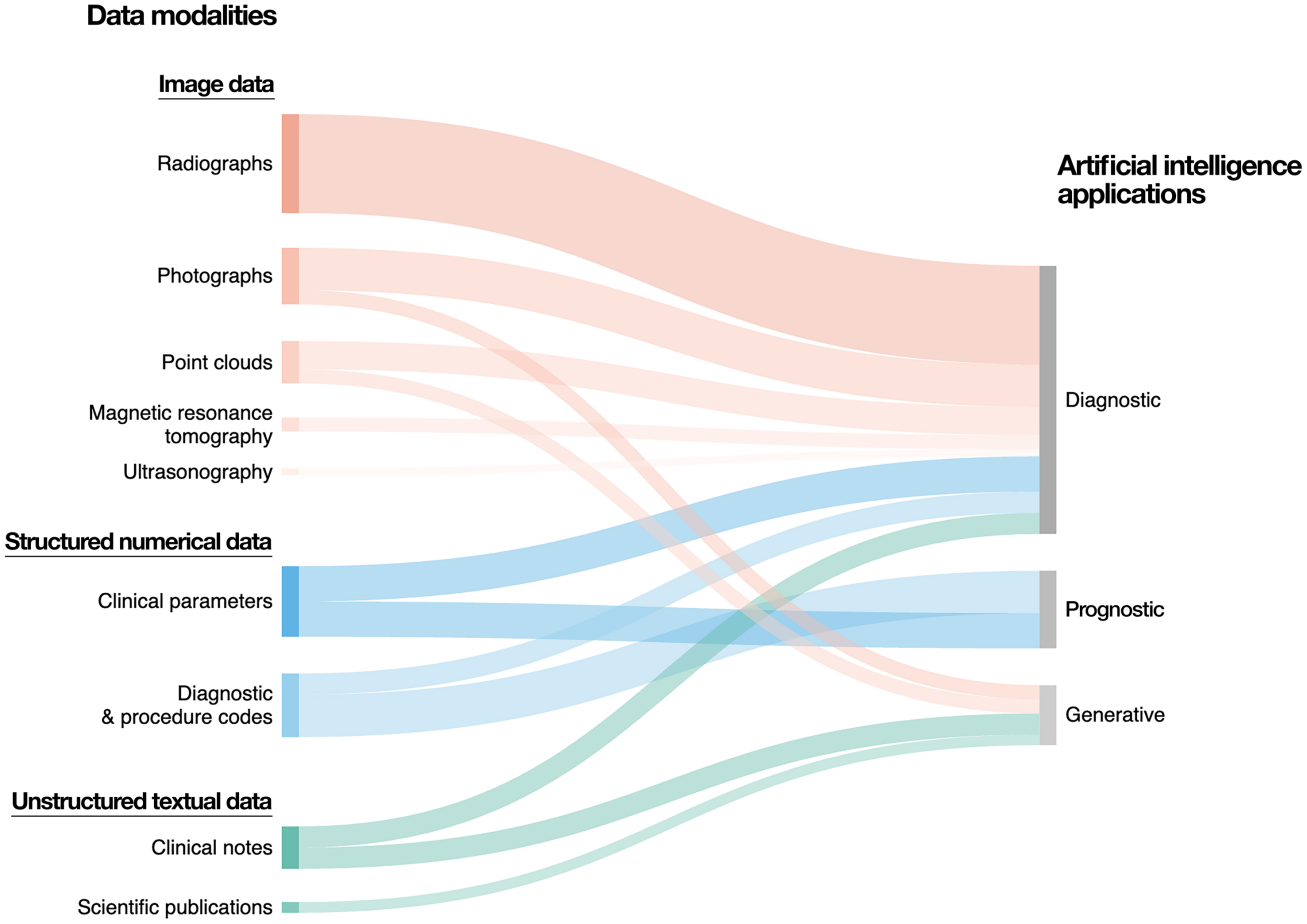
Data modalities and artificial intelligence applications in dental medicine. The majority of data generated in dental medicine can be categorized into image data, structured numerical data, as well as unstructured textual data. Image data predominantly includes radiographs, which can be two-dimensional (e.g., panoramic and periapical radiographs, bitewings) or three-dimensional (e.g., computed tomography scans). Further sources of image data include photographs and three-dimensional point clouds (e.g., intraoral scanning). Structured numerical data include demographic data, electronic dental records, as well as clinical parameters (e.g., periodontal probing depth, vertical alveolar bone loss). Unstructured text data include free-form clinical notes, patient testimonials, referrals, treatment plans, as well as academic publications and regulatory documentation.

The amount of electronic health data generated globally is sharply increasing (Favaretto et al., 2020; Schwendicke and Krois, 2022). In dental medicine, this data can be divided into three major overarching modalities: image data, structured numerical data, and unstructured textual data (Figure 2).

Image data include radiography, photography, ultrasonography, near-infrared light transillumination, histology, and three-dimensional point clouds. Arguably the most prevalent form of imaging in dental medicine, dental radiography ranges from small, two-dimensional intraoral images (e.g., intraoral periapical radiographs, bitewings) to large, three-dimensional cranial computed tomography (CT) scans. Although still considerably less prevalent than CT, magnetic resonance tomography is increasingly used as a radiation-free alternative, especially in larger hospital settings. Intra- and extraoral photography is an important diagnostic and documentary tool in oral medicine. Dental ultrasonography is a noninvasive, real-time imaging method surging in popularity after the development of small probes suitable for intraoral use (Chan et al., 2018). Histological imaging in oral and maxillofacial pathology is routinely used to assess tissue biopsies, comparable to general pathology. Three-dimensional point clouds are generated through intra- and extraoral scanning. Image data constitute a large share of all data created in dental medicine, largely owing to the high number of radiographs. Notably, the number of radiographs created in dental medicine is much higher when compared to other medical disciplines. Dentistry generates approximately 1.1 billion radiographs annually, which account for 26% of all radiographic procedures worldwide (United Nations Scientific Committee on the Effects of Atomic Radiation, 2022).

Structured numerical data (e.g., clinical parameters measured for clinical care and research, dental insurance claims, billing records) require standardized entry at least on the provider level. A considerable share of numerical data in dental medicine is either created by research institutions for clinical studies (i.e., patient-feature matrices) or through the billing of the rendered dental services (e.g., procedure codes). In addition to being assessed by clinicians, numerical data can also be self-reported (e.g., through forms or surveys) or even automatically measured (e.g., using wearable sensors). However, some sources of structured numerical data regularly registered in other medical disciplines (e.g., serum- or saliva-based diagnostic tests) might not be captured as a part of routine dental care.

Unstructured textual data (e.g., clinical notes) are widespread in electronic dental records (EDRs) from dental clinics and offices. Most clinicians do not maintain numerical data in a structured manner within the EDR for future reference, instead including numerical data (e.g., working length of a root canal, clinical attachment level measurements) within freeform clinical notes. In a broader sense, unstructured textual data can include patient and vendor correspondence, journal articles, and other written material. Inevitably, unstructured textual data represent the data modality most contingent upon its author; while image or numerical data can vary among providers due to differences in quality or calibration, textual data further depends on personal writing style.

Considering the unique advantages of each data modality, their integration plays a key role in rendering comprehensive dental care. Notably, most dental patients see their providers regularly and over longer periods of time. Put into the context of ML, this yields rich sources of longitudinal, multimodal datasets (radiographs, EDRs, clinical parameters, etc.). When used in ML, multimodal datasets allow for more accurate, robust data that include various aspects of an individual’s clinical and treatment history, creating a more personalized, integrated model.

## 2 Diagnostic modeling

Diagnostic modeling typically involves recognizing structures and patterns in the input data or classifying these into largely predefined categories (e.g., health or disease). In dental medicine, these input data are predominantly visual, with structured numerical data playing a secondary role. Imaging methods require visual assessment by the observer; with increasing complexity, the diagnostic difficulty increases. In addition, human diagnostic accuracy is inherently a function of the observer’s experience and attention, as well as the prevalence of the diagnosis. To mitigate these limitations, computer-aided diagnostics use ML and computer vision to aid the human diagnostic process.

### 2.1 Computer vision

**Figure 3:**
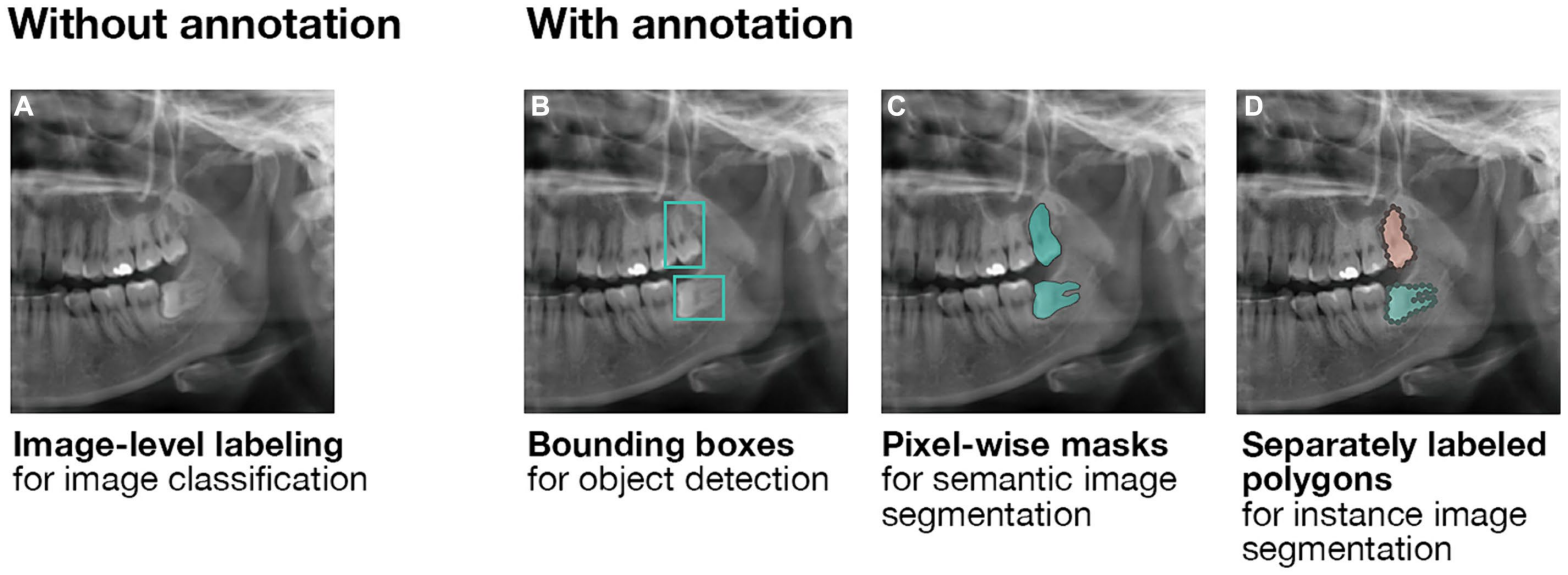
Labeling methods for computer vision. **(A)** Image-level labeling without further annotation enables the classification of entire images. **(B)** Annotating images using bounding boxes enables the training of object detection models. As the output of an object detection model is a predicted bounding box, it is unsuitable for the precise demarcation of anatomical structures. Here, third molar teeth are roughly localized. **(C)** Pixel-wise masks enable the training of semantic segmentation models whose outputs are pixel-wise masks as well. Importantly, no differentiation is made between multiple instances of the same structure. Here, all occurrences of a third molar are marked without differentiation between them. **(D)** Separately labeled pixel-wise masks enable further distinction between multiple instances of the same structure using instance segmentation models. Here, in addition to all occurrences of a third molar being marked, the two third molars are clearly distinguished from one another.

Computer vision refers to the inference of patterns from visual data (Esteva et al., 2021). To enable processing of the images by an ML model, a neural network fractionates images into single labeled pixels; convolutional neural networks (CNNs) are used for single images, while recurrent neural networks (RNNs) process a series of images (e.g., sequential images or videos). The neural network then uses the pixel-level labels to perform convolutions (i.e., a mathematical operation on functions f and g that produces a third function, f∗g ) and predict the content of the input data; the number of parameters to learn is reduced by down-sampling (e.g., pooling). This process’s accuracy depends on many factors, not least of which the chosen model and labeling method. Various labeling methods are demonstrated on a partial panoramic radiograph in Figure 3. At the basic level, image-level labeling enables classification of the entire image, without further separation of its contents (Figure 3A). Recognizing structures within images requires more detailed annotation. Bounding boxes (i.e., minimum enclosing rectangles) represent the simplest way to annotate a structure within a radiograph. While they can be quickly added to the image, they specifically enable basic object detection: the trained ML model coarsely localizes the structure, without accurate demarcation of its boundaries (Figure 3B). Meanwhile, pixel-wise masks assign a label to every annotated pixel. While the annotation process is considerably more labor-intensive, it enables the precise segmentation of an object (Figure 3C). For three-dimensional point clouds, point-wise masks are used analogously. A faster alternative to pixel-wise masks is the use of polygons, where the annotator fits geometric figures to the structure. Notably, masks and polygons can be assigned to different instances of the same class, enabling discrete segmentation of each instance (e.g., 18 and 28 are both third molars, but they are not the same third molar, Figure 3D).

The reliance on radiographic imaging for dental diagnostics and the consequently large amount of available radiographic data makes computer vision one of the most relevant areas of ML in dentistry. Using computer vision to detect and segment anatomical structures (e.g., teeth) enables automated dental charting (Gao et al., 2022; Kabir et al., 2022). Dental caries and periodontitis, two of the most prevalent pathological conditions worldwide (Tonetti et al., 2017; Bernabe et al., 2020), are diagnosed using radiographs as well as visual and tactile examination. However, radiographic diagnostics remain highly variable among examiners (Akesson et al., 1992; Bader et al., 2001). Diagnostic sensitivity is especially low for early caries lesions (Schwendicke et al., 2015). Computer vision is increasingly used to aid or automate radiographic diagnostics. In addition, there is an apparent shift toward more sophisticated segmentation models as opposed to simpler object detection models (Arsiwala-Scheppach et al., 2023). Compared with human observers, computer vision yields a higher sensitivity while maintaining noninferior specificity (Schwendicke et al., 2021b). Notably, the accuracy of caries detection increases nonlinearly with an increasing size of the training dataset (Schwendicke et al., 2022). Various computer vision applications have been proposed for the diagnosis of gingivitis and periodontitis (Revilla-León et al., 2022). In the radiographic detection of periodontal bone loss, computer vision does not appear obviously superior to human observers, mostly owing to inconsistent reporting quality (Patil et al., 2023). Periodontal diagnostics routinely involve the use of intraoral radiographs of the entire dentition. Compared to panoramic radiographs, intraoral images only capture a portion of the dentition and jawbones. The diagnostic workflow thus includes the mounting (i.e., correct arrangement) of these smaller radiographs. Applying image classification to entire intraoral radiographic series has been proposed as an approach for automating the mounting process (Lin et al., 2023).

While radiographic data still represent the largest portion of training data for computer vision in dentistry, models are increasingly trained on other relevant sources of imaging data. Clinical dental practice increasingly involves the use of intra- and extraoral photography as well as scanning which create two-dimensional RGB images and three-dimensional point clouds, respectively. These data sources enable novel uses of computer vision. Trained on intraoral photographs, CNNs can diagnose carious lesions (Moharrami et al., 2023), tooth crowding (Ryu et al., 2023), as well as dental anomalies associated with orofacial clefts (Ragodos et al., 2022). The classification of entire intra- and extraoral photographs has further been proposed as a way of automating the sorting and archival of orthodontic images for documentation and treatment monitoring purposes (Li et al., 2022). Trained on intraoral scans, CNNs enable instance segmentation of individual teeth (Zanjani et al., 2021; Vinayahalingam et al., 2023). Multimodal data consisting of point clouds and radiographic imaging further enable CNN-based superimposition of intraoral scans and cone beam CT images, given they are noninferior to human operators (Ntovas et al., 2024). Extraoral scans can be used to train CNNs to automatically locate orthodontic soft tissue landmarks (Baysal et al., 2016) as well as predict morphological changes following orthognathic surgery (Tanikawa and Yamashiro, 2021). While dental ultrasonography is a novel imaging method whose adoption remains low, CNNs have been successfully trained to identify anatomical structures on sonograms (Nguyen et al., 2021b).

To a large extent, computer vision used in dentistry has concentrated on individual models and tasks as opposed to more complex, multi-step workflows (Schwendicke and Krois, 2022). Some models can simultaneously detect and classify structures on radiographs (Yang et al., 2020). Notwithstanding the efficacy of single-step architectures, their nature is somewhat in contrast with clinical diagnostics where the human observer often performs a sequence of tasks to make a diagnosis. The process of inferring from the observer’s own previous experience as well as contextual information has been described as clinical diagnostic reasoning. To replicate individual steps of the human clinical diagnostic reasoning process, composite ML workflows of multiple models have been proposed (Feher et al., 2022). While these methods offer good explainability, as they closely resemble clinical reasoning, it is crucial to note that precisely emulating the stepwise diagnostic process of a human clinician does not result in superior diagnostic performance when compared to end-to-end approaches.

### 2.2 Classification

Patterns in structured numerical data have been used for automated diagnostics based on classification since the 1970s (Leonard et al., 1973, 1974). From a technical standpoint, the modeling architectures for classification and prediction tasks are comparable. The term “prediction” itself can broadly refer to the output of any ML model (e.g., an object detection model “predicts” a bounding box). Essentially, classification determines categories for elements already within the dataset whereas prediction approximates elements missing from the dataset. Clinically, the distinction lies in whether the ML model’s output exists at the time of application: classification mainly establishes a present diagnosis (e.g., disease staging) whereas prediction can also estimate potential future events (e.g., risk of complications).

Classification algorithms used in dentistry are typically trained on labeled data. In addition to CNNs and related neural networks, several supervised non-deep learning approaches are routinely used. These include decision trees and Random Forests, Support Vector Machines, Bayesian networks, K Nearest Neighbors, and Voting Feature Intervals classifiers (Singh et al., 2016; Joshi and Jetawat, 2020). Random Forests and Support Vector Machines represent the most prevalent non-deep learning dental classification approaches, each operating upon a different base architecture and specialized in modeling different scenarios (Arsiwala-Scheppach et al., 2023).

Outside of computer vision, structured numerical data represent the primary data modality for classification models. Data derived from clinical examinations and radiographs can be used to train ML models that classify patients’ maxillofacial morphology (Ueda et al., 2023), establish primary orthodontic diagnoses, assess treatment need and support treatment planning (Thanathornwong, 2018; Prasad et al., 2023; Senirkentli et al., 2023). An advanced application of ML-supported treatment decision-making based on classification modeling involves the planning of removable partial dentures (RPDs) and the subsequent ability to autonomously generate customized RPD designs. Although this might resemble generative modeling, the underlying architecture actually classifies input structures into predefined Java curve functions, the collective arrangement of which constitutes the design for an RPD (Chen et al., 2020). Importantly, unstructured data cannot be processed natively by classification algorithms, thus the information contained therein remains unusable for classification purposes.

### 2.3 Natural language processing

Dental medicine generates a large body of unstructured language data in the form of clinical notes (Pethani and Dunn, 2023), published research articles, clinical correspondence, and verbal transcriptions. Natural language processing (NLP) is a branch within AI that focuses on using models to understand, analyze, and structuralize human language; this applies to both written and spoken text. The application of NLP in medicine is highly logical, given that clinicians compile extensive patient information in unstructured text (Jensen et al., 2017; Sheikhalishahi et al., 2019). The application of NLP to extract information from clinical text is thus increasingly relevant (Kreimeyer et al., 2017; Wu et al., 2019). Named entity recognition and relation extraction, NLP techniques that identify key entities and their relation from text corpora, have demonstrated high precision and recall when extracting information from medical text (Li et al., 2023; Raza and Schwartz, 2023).

In dentistry, NLP has a wide range of potential use cases (Büttner et al., 2024); the most obvious one is presently the analysis of EDRs. NLP can be used to structure free-text EDRs to extract information pertaining to patients (e.g., prior clinical diagnoses) as well as previous treatments (e.g., previous restorative procedures, materials used, etc.) (Chen et al., 2021; Patel et al., 2022a). Analyzing longitudinal EDR data using NLP further enables the tracking of temporal changes, as shown in previous work monitoring periodontal disease progression (Patel et al., 2023b).

Compared to text corpora, the processing of spoken words involves the additional step of translating the audio signal; deep neural networks can be used to support this process (Nassif et al., 2019). Applied to the transcripts of clinical case vignettes, NLP shows noninferior dental charting performance compared to human experts (Zhang et al., 2021). NLP, coupled with speech recognition, thus shows high potential to increase administrative efficiency.

In addition to clinical practice, NLP has potential in dental education, with success identifying clinically relevant messages from a large dental online community forum (Bekhuis et al., 2011). Recent trends in the United States (US) show a decline in practice ownership among dentists, along with a growing tendency for new graduates to join dental service organizations; meanwhile, only a small fraction of graduating dental students envision a career in academia (Istrate et al., 2023). Outside academia, clinicians can use discussion groups, forums, or social media to acquire and exchange insights. As NLP has been shown to effectively extract relevant messages from online dental discussion groups, it can potentially aid clinicians in sifting through information and experiential cases from their peers (Bekhuis et al., 2011).

### 2.4 Limitations

While AI has already demonstrated potential in diagnostic modeling across computer vision, classification, and NLP tasks, limitations exist regarding data quality and quantity, explainability, generalization, cost and infrastructure, as well as clinical validation. As diagnostic modeling is arguably the most important area of applied AI in medicine, these limitations are particularly important. Indeed, most studies on either computer vision or other classification models are limited by single-center datasets. AI’s diagnostic capabilities rely on the datasets used to train the model; consequently, the diagnosis will be biased toward the outcomes of the training set and will not account for potential variability between different patient populations. This pertains to the diversity of patients in the training sets, the accuracy of the labeling of the data, and the quality of the data itself. The inherent homogeneity of patients collected within a single clinical practice exacerbates this problem, and cross-center validity remains a challenge (Schwendicke et al., 2021a). Indeed, clinical validation requires the rigorous testing of AI-enabled tools against large, diverse datasets. Further constraints of certain diagnostic methods lie in the heterogeneity of reported performance metrics, as well as the models’ explainability and clinical applicability. The necessity for human supervision is presently a non-negotiable aspect of any clinical encounter, and transparency is of utmost importance in the decision-making process. Nonetheless, the utility of AI-enabled tools as clinical decision support systems still presupposes the interpretability of their predictions and recommendations (Kundu, 2021; Reddy, 2022).

## 3 Prognostic modeling

While diagnostic modeling focuses on identifying existing conditions, ML models can also be trained to anticipate future events in a proactive manner in the form of prognostic modeling. Patterns identified in large longitudinal datasets can be extrapolated into the future, thereby estimating events like the onset of diseases, success rates of performed procedures, and epidemiological trends. In dentistry, prognostic modeling can be especially valuable for preventive care and early diagnosis to deliver more personalized, holistic clinical care.

**Figure 4:**
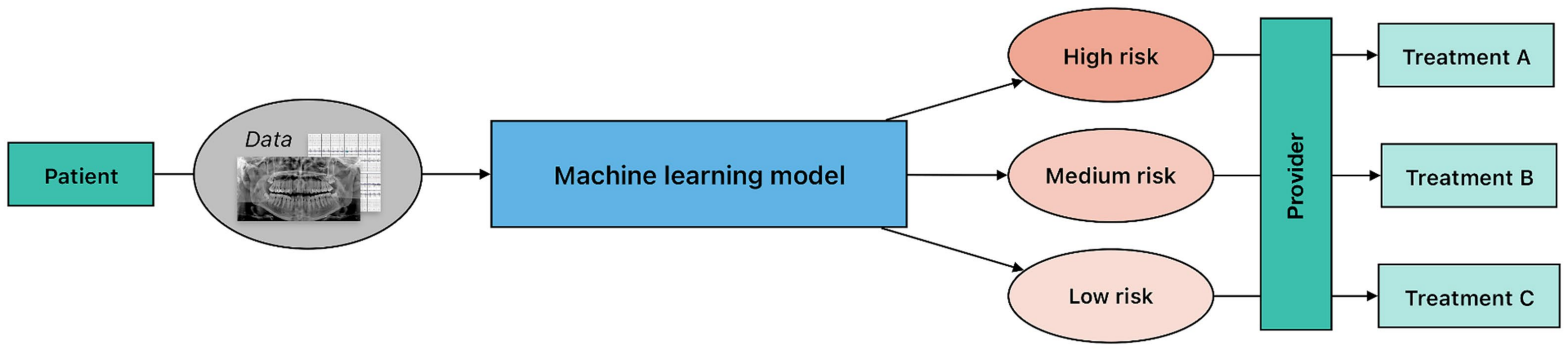
Overview of a clinical risk stratification workflow. In this process, predictive modeling is applied to patient data to classify individuals into predefined risk categories. Notably, although the underlying machine learning architecture may be similar to diagnostic modeling, risk stratification predictions lack a definitive ground truth. The provider uses the information from the model to guide the treatment planning and risk prediction at the patient level.

Prognostic modeling uses various algorithms to forecast outcomes based on a set of inputs. Discriminative models, or models that learn P(Y|X) , are particularly effective for classification tasks with discrete outputs. These models use training data to learn the boundaries between classes and then utilize the learned boundaries to predict a class, or a forecast, from a given input; for example, using input data like age, past medical history, past dental history, diagnostic biomarkers combined with other risk factors to classify individuals into categories of periodontal disease risk (low, medium, high) (Figure 4). Regression models are also widely used for prognostic modeling, particularly for continuous outputs. Regression is a form of supervised ML that aims to create a “best fit” line or curve to best describe the relationship between independent (input) and dependent (output) variables. This “best fit” model is then used to predict outputs given a set of new, unseen inputs.

In addition to regression modeling, classification models can also be used for prognostic tasks, including Random Forests, Support Vector Machines, and neural networks (Arsiwala-Scheppach et al., 2023). Random Forests and Support Vector Machines are architectures mainly used for classification tasks, the latter focusing on the data points closest to the decision boundary by finding an optimal separating hyperplane. Neural networks function by possessing a network of nodes, referred to as neurons, that are connected based on weighted paths.

Temporal changes in parameters can be used to identify patterns more accurately through the use of longitudinal data. RNNs were designed to manage sequential data, enabling them to consider temporal changes. RNNs can further be enhanced using intra-attention mechanisms to emphasize the portion of sequences contributing the most to predictions (Bahdanau et al., 2014; Luong et al., 2015). In addition, RNNs can be enriched using different types of layers, including CNNs and graph neural networks (Ma et al., 2020; An et al., 2021).

Structured numerical data, the primary data modality for prognostic modeling, have been used to train prediction models for tooth loss (Hasuike et al., 2022), periodontitis (Patel et al., 2022b), peri-implantitis (Mameno et al., 2021; Fan et al., 2023), xerostomia (Lee et al., 2024), Sjögren’s disease (Mao et al., 2024), medication-related osteonecrosis of the jaw (Kim et al., 2018), the malignant progression of oral leukoplakia (Liu et al., 2017), as well as the survival of patients with oral squamous cell carcinoma (Kim et al., 2019). Further, prediction models based on structured numerical data have been proposed for the prognosis of certain dental treatments, including root canal treatment (Bennasar et al., 2023) and dental implants (Ha et al., 2018; Rekawek et al., 2023). As previously mentioned, structured numerical data can be collected from patients in a self-reported manner, as shown in previous work on predicting tooth mobility (Yoon et al., 2018).

Notably, AI can be used to obtain structured numerical data from other data modalities. Prediction models for postoperative neuropathy after mandibular third molar extraction showed noninferior performance when human observers evaluated native three-dimensional radiographs compared with STL files pre-processed with computer vision for anatomical segmentation (Picoli et al., 2023). Crucially, the prediction models still relied on structured numerical data compiled by human observers from the image assessments.

### 3.1 Limitations

While prognostic modeling is not the predominant application of AI in dental medicine, estimating future risk and identifying patients at risk for diseases and complications is highly valuable for its clinical usefulness. However, a considerable distinction exists between diagnostic and prognostic modeling in the realm of trust. While the output of a diagnostic model can be immediately compared with other diagnostic tools to verify a diagnosis, a prognosis cannot be immediately validated. This increases the necessity of trust in the AI model’s forecast and underscores the importance of predictive accuracy and model calibration: miscalibration of the model can lead to overtreatment if a patient is incorrectly deemed at risk or, conversely, serious health issues if a risk is underestimated (Van Calster et al., 2019). This emphasizes the critical importance of AI interpretability, which remains the primary challenge in predictive modeling to eliminate the one-size fits all approach that exists in many facets of dental medicine (Giannobile et al., 2013a,b). Emerging diagnostics are creating expanded opportunities for clinicians to combine saliva diagnostics and chairside clinical innovations to advance precision care that can be advanced with these approaches (Steigmann et al., 2020).

Practically, the biggest limitation standing between clinical practice and prognostic models is the reliance on structured data. During a clinical trial, information is recorded into a patient-feature matrix, which makes the data readily accessible to algorithmic processing, including the training of ML models. In contrast, practicing clinicians routinely store information, including numerical measurements, in free-form clinical notes. Even though most health records are digitized, clinical notes are generally only comprehensible to humans. This limits not just the training of new prognostic models based on data routinely gathered through clinical practice, but also the application of existing prognostic models to patients outside of clinical study settings. To maximize patient benefit from AI-enabled tools, their applicability in clinical practice needs improvement. A crucial part of this improvement is making data progressively machine-readable.

## 4 Generative modeling

**Figure 5:**
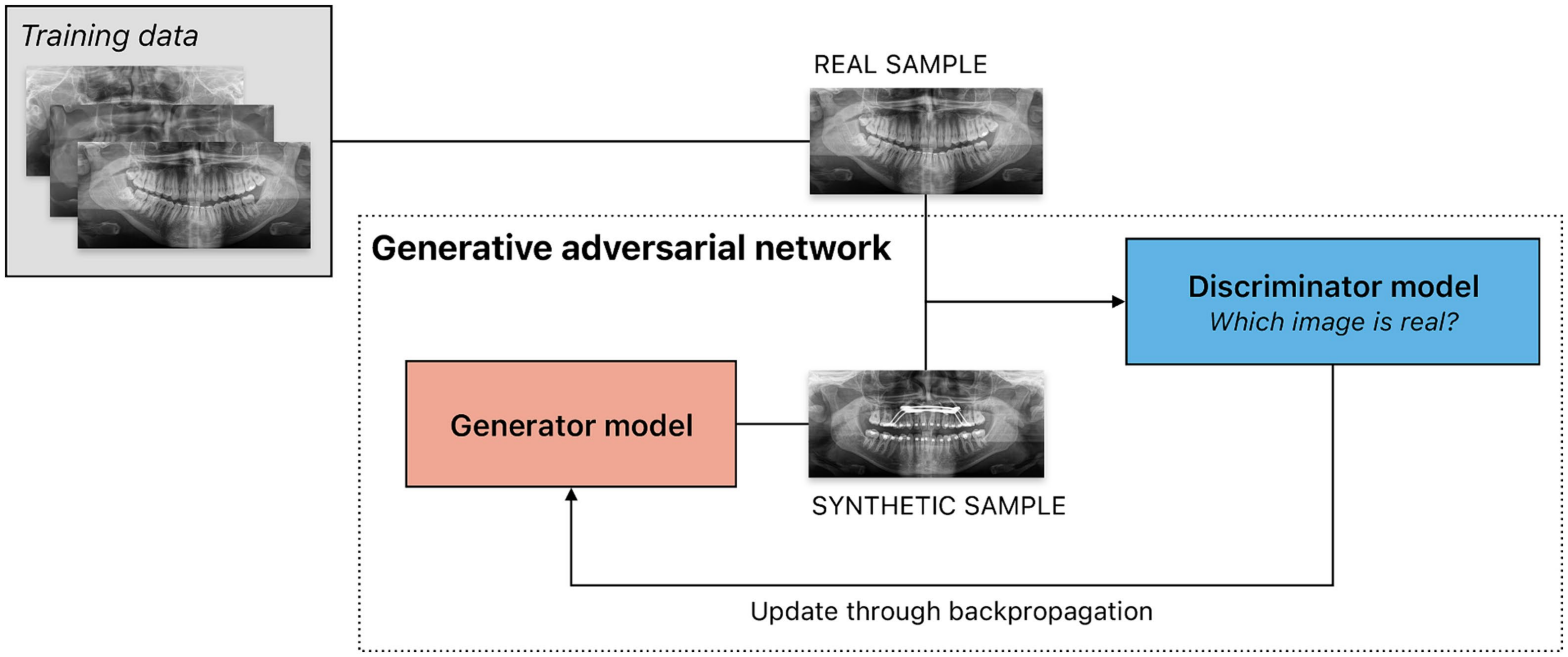
Sample scenario illustrating the use of a generative adversarial network (GAN) to create synthetic panoramic radiographs. GANs consists of two models, a generator and a discriminator, trained simultaneously through an adversarial process. The generator is tasked to create synthetic data intended to mimic real data — in this example, panoramic radiographs. The discriminator is a classifier that evaluates input from the generator as well as the training dataset, attempting to determine which are real or synthetic. The result of the classification task — success or failure — is used as feedback for both models. The generator thus aims to create data indistinguishable from real data to the discriminator, while the discriminator aims to distinguish generated data from real data. Feedback from the discriminator is used to train the generator. Through this iterative adversarial process, the generator improves at producing realistic data, while the discriminator improves at classifying data as real or synthetic.

Generative models learn the probabilistic distribution of the input space P(X) and use these probabilities to generate new data. Often in response to prompts (i.e., descriptions of the AI task in natural language), generative models create synthetic new data (e.g., text, images, audio, video) with similar characteristics to their training data. Numerous generative models each employ a distinct method for content creation. The most commonly used approaches include generative adversarial networks (GANs), transformer-based models, and diffusion models. A sample approach to generate synthetic radiographs using a GAN is demonstrated in Figure 5. Transformers build the basis of most contemporary large language models (LLMs), artificial neural networks for general-purpose language creation.

Synthetic data produced by generative models can be useful for model training in research or educational purposes without compromising patient privacy (Umer and Adnan, 2024). Further, synthetic data can reference real information. Through generative summarization, LLMs can synthesize overwhelming amounts of electronic health data and create a clinical narrative summary (e.g., for the automation of clinical notes after a patient visit). Although the generation of dental-specific notes has yet to be detailed in scholarly work, summarization models have demonstrated success in creating clinical notes based on structured health record data from electronic health records (Gong and Guttag, 2018). LLMs have further been shown to generate discharge summaries (Patel and Lam, 2023) and correspondence to patients detailing their diagnoses and post-operative instructions (Ali et al., 2023). Notably, in some instances, health care professionals have been shown to prefer LLM-generated responses to questions asked by patients in an online forum over physicians’ answers in both information quality and empathy (Ayers et al., 2023). Current results from oral and maxillofacial surgery further suggest a higher quality of responses to patient questions than to technical questions asked by surgeons (Balel, 2023).

In addition to generating text corpora through LLMs, generative AI holds significant potential in creating synthetic images for settings where images from real patients cannot be utilized. Generative models, for example, have been shown to produce intraoral images that were, at a resolution of 512 px ∗ 512 px, indistinguishable from real images in a pediatric dental setting; rough artifacts remained visible at a resolution of 1,024 px ∗ 1,024 px (Kokomoto et al., 2021). The use of diffusion instead of GAN for image creation can potentially improve this limitation, as diffusion models have been shown to outperform GAN models on all tasks (Saharia et al., 2022). Still, GANs can achieve performance satisfactory for clinical application, including the generation of crown designs from point clouds obtained from intraoral scanning (Ding et al., 2023).

**Figure 6:**
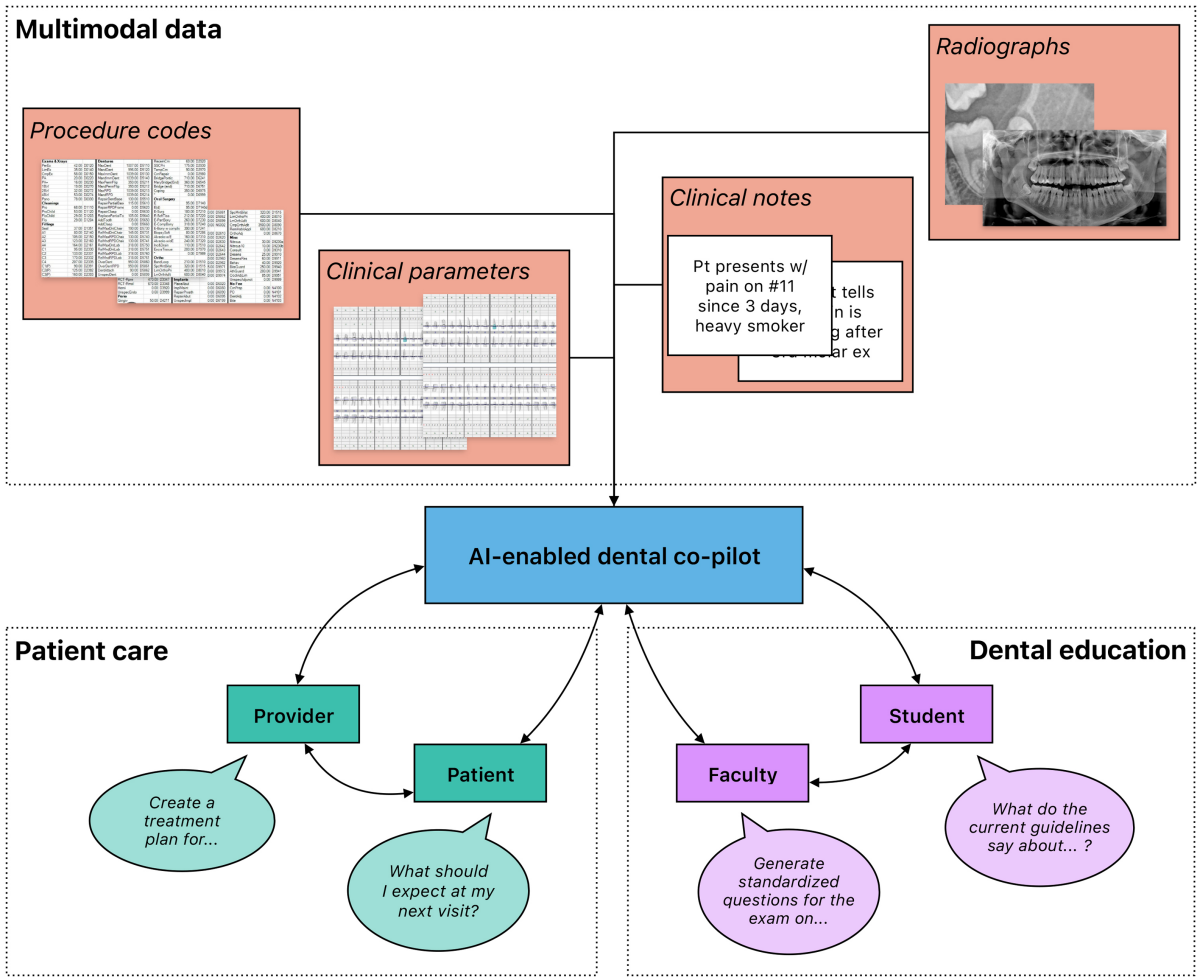
High-level overview of the potential use of generative AI in dental medicine. Rich, multimodal data gathered from clinical practice and dental research could be leveraged to train generative AI models that function as co-pilots to dental professionals. These models could be deployed in a variety of different settings, including patient care and dental education. Importantly, multiple stakeholders benefit from their interactions with the models. In patient care, both providers and patients could leverage generative AI to gain information; providers could further automate administrative processes to increase efficiency. In dental education, faculty and students could both engage with generative AI to create an immersive learning environment with individualized feedback.

Besides research and practice, AI shows potential in medical education as well. LLMs have been shown to create viable, high-quality multiple-choice questions when given adequate guidance (i.e., question difficulty, number of questions, and answer choices) with prompt engineering (Johnson et al. 2023). While specific questions from the study still required improvement following faculty review, this technology could improve the quality of didactic questions, reduce faculty workload, and minimize cheating through exam reuse over time. Based on the performance of contemporary LLMs in other areas, it further seems plausible to retrain or fine-tune models with data relevant to oral health education and create a virtual patient with which students and educators can interact to enable learning in an interactive, immersive manner (Figure 6).

### 4.1 Limitations

Given how a generative AI model functions on probabilistic distributions, guessing the next token in a sequence through auto-regressive prediction, hallucinations (i.e., fabricated information presented as factual) present one of the most severe limitations. In the medical domain, hallucination has been demonstrated through asking an LLM to explain the pathophysiology of osteoporosis; none of the five articles referenced by the model existed, and all had PubMed IDs that were of different, unrelated papers (Alkaissi and McFarlane, 2023). Hallucinations are especially dangerous in the clinical context, where erroneous information can place patients at risk. It remains uncertain whether hallucinations can be prevented through gradual improvements in the existing LLM architecture, or if a deeper fundamental transformation in the LLM methodology may be necessary to prevent nonfactual responses. Creating an LLM that must provide citations for factual responses could be a potential solution (Glaese et al., 2022). Adjusting the prompt fed into the LLM with question-specific context has also proved successful (Feldman et al., 2023). The temperature of a model, a hyperparameter that can be fine-tuned by the engineer, controls how “random” the output of a generative model is; a higher pre-programmed temperature increases the chance of the model following a word-path with a lower initial probability, and consequently increases the risk of hallucinations. In dentistry, where hallucinations pose such large threats for patient care, a lower temperature parameter is essential.

Generative models are inherently reliant on the data used to train them. There are limitations both in finding sufficient, reliable training data and in the biased output that the training data can produce. In dental medicine, protecting patient privacy is paramount (Joda et al., 2019). When using generative models, the user must be aware of where the private patient data they input into a model are being stored. Commercially available LLMs might store user interaction data on their servers unless a user chooses to opt out (OpenAI, 2023a,b). Utilizing closed-sourced LLMs that are run by private companies means that potential patient data sent to the model will be sent to the company’s server, which may not be compliant with data privacy regulations. This limitation could be solved using open-sourced models that can be downloaded to run on an individual’s private server, one that can be compliant and already stores critical patient data. The unique characteristics of LLMs require a tailored, adaptive approach to regulatory oversight to ensure ethical use (Meskó, 2023). Further, the data used to train generative models must be stripped of patient identifiers, as the use of traceable information when outputting new responses must not be risked. Because of this risk of using identifiable data, acquiring sufficient and robust training data to produce accurate, reliable models is extremely difficult. Alternatively, data can be sanitized of sensitive or personally identifiable information, but the process is rigorous and reliant on human error and time. Moreover, even when training data are acquired and sufficiently sanitized, there is a risk of a biased output dependent upon biased training data. If only data from a certain population demographic or location are used to train the models, not only will the output be inherently biased, but it can consequently propagate the underlying bias. The difficulty of acquiring sufficient, accurate, robust training data increases the risk of engineers becoming less strict about the population and any clinical bias the data may possess. In the medical domain, it is of critical importance to ensure that the data used applied ML models are representative of the entire population.

## 5 Future considerations and directions

Adopted by all stakeholders in a fair, ethical, transparent, and equitable manner, the application of AI has transformative potential in dental research and clinical care (Table 1). For clinicians, application of AI in dental medicine can enhance diagnostics, treatment planning, and outcome prediction. For providers, AI can improve operations, decision support, and case acceptance. For patients, AI can increase diagnostic transparency and enable more efficient treatment monitoring. In the optimization of administrative tasks and the generation and implementation of synthetic data in oral health education, AI can streamline workflows, reduce administrative burdens, and provide personalized patient care. To this end, explainability and generalizability of ML models used in dentistry must be maximized. Datasets used for training should be diversely sourced and validated across patient populations; single-center datasets should only be used for proof-of-principle reports or if there is a specific reason in connection with the research question. Ideally, both datasets and codebases should be made openly available to other researchers to enable thorough peer review and reproducibility. Transparency and interpretability are key desiderata for the application of AI in any medical setting.

**Table 1:** Opportunities to apply artificial intelligence in dentistry [adapted from Elani and Giannobile (2024)].

Stakeholders	Opportunities to apply artificial intelligence
Dental clinicians	Assisted diagnostics with automated documentation Patient education Assisted treatment planning Treatment outcome prognostication
Clinics and providers	Scheduling, billing, and reimbursement optimization Patient triage and management through virtual assistants Clinical decision support
Patients	Transparency in diagnostics and patient information on treatment need Continuous treatment monitoring
Insurers and payers	Optimized operations (e.g., prior authorization) Automated administrative workflows Fraud prevention
Academic institutions	Education and training on artificial intelligence Synthetic patient cases for personalized dental education Utilization of longitudinal datasets for research
Regulatory bodies	Framework for fair, transparent, and equitable use of artificial intelligence Ensure compliance of other stakeholders

The clinical use of AI is subject to medical device regulation. While this process is generally administered at the national level, supranational unions with specialized medical regulatory agencies streamline regulation for large populations: the European Medicines Agency of the European Union (EU, population: 450 million), the Medical Device Committee of the Association of Southeast Asian Nations (ASEAN, 670 million), and the forthcoming African Medicines Agency of the African Union (1.3 billion). Notably, supranational unions allow their members to regulate on the national level (e.g., the Health Services Authority in Singapore, an ASEAN member state). In stark contrast, regulation is strictly kept at the national level by some of the largest countries in the world by population, including India (Central Drug Standard Control Organization), China (National Medical Products Administration), and the US (Food and Drug Administration, FDA). The FDA has no distinct clearance process for AI-enabled products and a large share obtains regulatory clearance through the existing 510(k) premarket notification pathway, arguing substantial equivalence to a former FDA-cleared device (i.e., predicate) (Joshi et al., 2024). However, recent data show that 33% of devices cleared through the 510(k) pathway were cleared on the basis of predicates whose first generations did not have any AI-enabled functions; for an additional 8%, it was unclear whether the first-generation predicates had any AI-enabled functions (Muehlematter et al., 2023). Contrary to the non-medical software industry which, for the most part, represents a global market, the landscape of medical device regulation has resulted in considerable fragmentation in the market of AI-enabled medical software. Inevitably, this prevents the clinical adoption of AI in dental care on a global scale: with developers incentivized to maximize their return on investment, many are discouraged by the challenges of navigating non-standardized global regulatory and insurance environments.

The quality of training data critically influences an AI model’s outputs. Moreover, the AI model will be reflective of the patient population utilized in its training. High-quality datasets that represent diverse patients across various locations, ages, backgrounds, and clinical situations in large volumes are necessary for developing robust models. Additionally, the quality of data labeling is vital to the model’s quality. Accurate annotation requires domain expertise and is time-intensive with large datasets. This necessity for extensive and diverse data is a limiting factor to model creation and consequently, AI innovation in dental medicine, due to the challenges in acquiring and accurately labeling such data.

Data privacy is integral to the implementation of AI in dental medicine. Data privacy regulations, which vary by country, impose stringent restrictions on the transfer, storage, and processing of patient data to protect privacy. Developers of AI-enabled medical software must ensure compliance with local regulations, each country having their own policies on how patient data can be utilized. For any organization operating in the EU or using the personal data of its citizens, the General Data Protection Regulation must be followed. In the US, the Health Insurance Portability and Accountability Act applies to any organization acting primarily in the US or dealing with US healthcare data. For AI research involving data sharing, a data use/sharing agreement is essential to specify the terms of exchange and ensure regulatory compliance. Compared to other areas, adherence to data privacy regulations inevitably throttles development of medical AI technology, as the stringent legal requirements render the acquisition of patient data complex and resource-intensive.

From the patient’s perspective, data privacy considerations include informed consent. Generally, patients can revoke consent for the inclusion of their data in biomedical research. However, this becomes impractical for large ML models trained on millions of patient records; removing individual records and retraining models is unrealistic if consent is revoked. Moreover, much of the data used for contemporary model training was collected retrospectively before medical AI became mainstream, meaning patients were unaware that their consent would effectively become irreversible. It is thus essential that patients be adequately informed if their data are used to train ML models.

Technologically, understanding the processes and potential pitfalls of many ML algorithms can be challenging due to their inherent lack of transparency from input to output. ML models often exhibit a “black box” nature, obscuring decision-making processes and complicating error detection. This issue is particularly acute in medicine where precision is paramount. Explainability, or the aim to address this “black box” ambiguity, is essential to have proper accountability, trust, technological accuracy, compliance, and continued model improvement as AI evolves.

From a global health viewpoint, achieving equitable AI implementation in dental medicine is essential. Evidence suggests that AI could reduce health disparities between high-income nations with comprehensive health care systems and lower-income countries (Ciecierski-Holmes et al., 2022; Elani and Giannobile, 2024). Nonetheless, this requires global cooperation on knowledge as well as data sharing to facilitate robust, multi-center ML development and implementation worldwide, thereby earning the trust of clinicians and health care professionals. Further, dedicated efforts (e.g., scaling programs) should be directed toward assisting lower-income countries to adopt AI-enabled solutions in health care, including dental medicine.

Taken together, a growing body of literature underscores the considerable potential of AI in dental medicine, while also highlighting obstacles that hinder the integration of AI-enabled tool into routine clinical workflows. Enhancing the transparency, interpretability, and reliability of these technologies, establishing robust regulatory frameworks, and providing support in underserved regions could pave the way for a future where AI enables better and more equitable patient care on a global scale.

## Author contributions

BF: Conceptualization, Data curation, Funding acquisition, Methodology, Visualization, Writing – original draft. CT: Conceptualization, Data curation, Funding acquisition, Methodology, Visualization, Writing – original draft. WVG: Conceptualization, Funding acquisition, Project administration, Supervision, Writing – review & editing.
